# The Impact of Payment Scheme Changes on Medication Adherence and Persistence of Patients Diagnosed with Depression in Korea

**DOI:** 10.3390/ijerph191711100

**Published:** 2022-09-05

**Authors:** Gyeongseon Shin, Bohwa Jang, Green Bae, Ha-Lim Jeon, SeungJin Bae

**Affiliations:** 1 College of Pharmacy, Ewha Womans University, Seoul 03760, Korea; 2 School of Pharmacy, Jeonbuk National University, Jeonju 54896, Korea

**Keywords:** individual psychotherapy, medication adherence, medication persistence, depression

## Abstract

As of 1 July 2018, the Korean National Health Insurance Service (NHIS) changed the fee schedule for individual psychotherapy (IP). We sought to analyze the impact of the IP payment scheme changes on the medication adherence and persistence of patients diagnosed with depression in Korea. We utilized the NHIS claims database from 2017 to 2019. Patients who were newly diagnosed with depression and utilized IP and were prescribed antidepressants during the study period were included. Adherence was measured using the medication possession ratio (MPR), and persistence was measured using the length of therapy (LOT) during the follow-up period. Adherence and persistence during the pre-policy period (before the change of the payment scheme, from January 2018 until June 2018) and the post-policy period (after the change, from July 2018 until December 2019) were compared. During the study period, a total of 176,740 patients with depression were identified. The average MPR significantly increased from 0.20 to 0.33 in the pre- and post-policy periods, respectively (*p* < 0.001). The average LOT of the patients improved considerably from 36 to 56 days in the pre- and post-policy periods, respectively (*p* < 0.001). Poisson regression analysis showed that patients with depression who were female, 19–34 years of age (vs. 50–64 years or over 64 years), and in the post-policy period were significantly associated with greater adherence and persistence rates. Payment scheme changes were associated with an increased adherence and persistence of medication use among patients diagnosed with depression.

## 1. Introduction

In South Korea, the prevalence of depression or symptoms related to depression in 2020 was 37%, which was the highest rate among the OECD countries [1]. Antidepressants are prescribed to treat depression [2], but non-adherence and early discontinuation of prescribed medication are prevalent [3,4]. Medication non-adherence poses a significant burden, such as increased suicide risk, hospitalization relapse, and added healthcare costs [5,6,7]. Etiologies behind medication non-adherence for patients with a mental disorder can be explained by patients’ perceptual factors and practical factors [8,9]. Patients’ negative attitude is the most critical barrier to medication adherence [10].

The importance of the clinician–patient alliance and clinicians’ active involvement in improving medication adherence has been reported previously [7,11,12,13]; through physician communication, patients are better informed about their diagnosis and the pros and cons of treatment [14]. Therefore, having sufficient time for the clinician–patient interaction should be the first step for medication adherence, yet 73.5% of individual psychotherapy (IP) sessions lasted less than 15 min in Korea [15], which might not be long enough to provide sufficient counseling for patients. The payment scheme in Korea is a fee-for-service system based on a resource-based relative value scale (RBRVS), and in order to encourage utilization of long sessions, the Korean government decreased RBRVS for short sessions and boosted RBRVS for long sessions as of 1 July 2018. Specifically, the new scheme reduced the fee for visits that lasted less than 10 min but almost doubled the fee for visits that lasted longer than 40 min (Appendix A Table A1). In addition, the patient co-payment for the IP sessions was reduced by 20%, such that if a patient received IP at the clinic, the financial burden would be less than it was previously.

With the introduction of the new payment scheme, we hypothesized that the number and length of IP sessions for patients would increase, which in turn would increase medication adherence or persistence among patients with depression. This study aimed to analyze the impact of the changed payment scheme in Korea on medication adherence and persistence among patients with depression by comparing patient behaviors before and after the introduction of the payment scheme.

## 2. Materials and Methods

### 2.1. Data Source

We conducted a retrospective cohort study from 1 January 2017 to 31 December 2019, using the National Health Insurance Service (NHIS) claims database. The NHIS claims database provides patients’ demographic characteristics, diagnosis codes (International Classification of Diseases—10th Revision (ICD-10)), the date of visit (claim), and pharmacy claim records, which include the number of prescriptions filled during the study period, the duration of the medication, and the prescribed drugs’ international non-proprietary names. Patient identification codes were removed to protect patient privacy [16,17].

This study was approved by the institutional review board of Ewha Womans University (protocol code ewha-202104-0030-01).

### 2.2. Study Population

**Figure 1:**
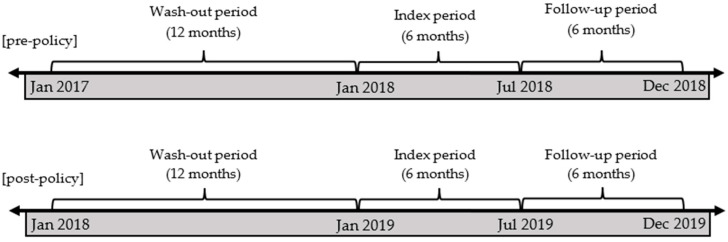
Study design.

**Figure 2:**
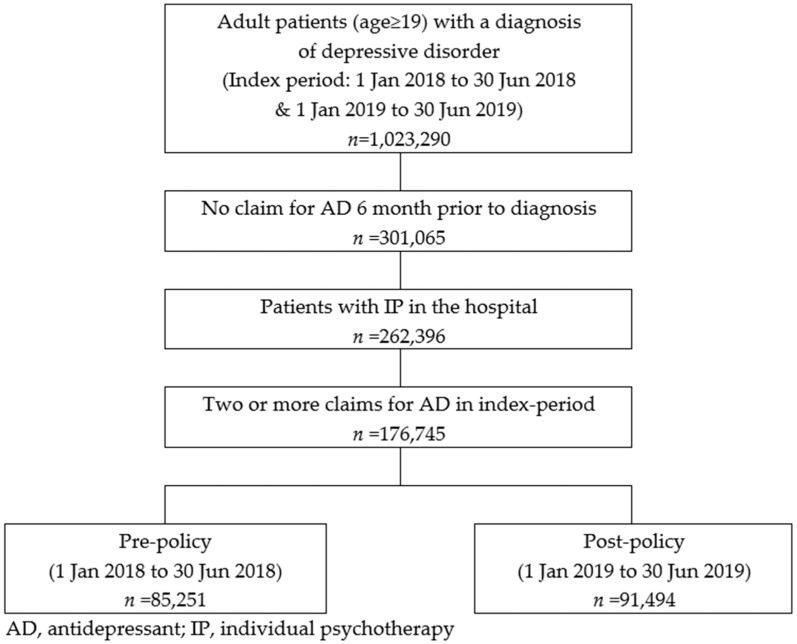
Summary of study design and population.

Individuals aged 19 or older who were primarily diagnosed with depression (ICD-10 codes F32–34 and F43) during the index period (from January 2018 until June 2018 for the pre-policy period and January–June 2019 for the post-policy period) were included. To exclude patients who had been previously diagnosed with depression, a 12-month washout period was used (Figure 1). Participants were also required to receive medication treatment and at least two claims for the antidepressants during follow-up (Figure 2). The first date of the diagnosis was used as an index date (i.e., the index period). A six-month follow-up period was used to measure adherence and persistence (Figure 1). The study design is summarized in Figure 2. The list of antidepressant drugs was referenced from the clinical practice guidelines [18,19] and is summarized in Appendix A Table A2.

### 2.3. Outcomes and Variables

The number of IP sessions was counted in the index period, and medication adherence and persistence were estimated in the follow-up period using the medication possession ratio (MPR) and the length of therapy (LOT), respectively. The MPR was defined as the number of days the patient possessed the study medication, divided by the observation period of 180 days [20,21]. An adherent patient was defined as an MPR being more than or equal to 0.8 [22,23]. The LOT, which measures the duration of time from the initiation to the discontinuation of therapy, was defined as the number of days from the index date to the earliest ending date of the last prescription, with the permissible gap being 14 days [23,24]. A patient was defined as persistent if the duration of uninterrupted use was at least 90 days [20,25].

### 2.4. Statistical Analysis

Descriptive statistics were reported as means and standard deviation (SD) for continuous variables and as frequencies for categorical variables. Chi-square tests were then used to compare the characteristics of the pre-policy group and the post-policy group by various demographic variables, such as sex, age, and the type of medications. The MPR and the LOT were compared using the *t*-test. The dependent variables in the univariate and multivariate analyses were adherence (measured by the MPR), and the key independent variable was whether the therapy occurred before or after the policy. Poisson generalized linear model (GLM) regression was used to evaluate relative risks and confidence intervals between the revised IP payment scheme and adherence. To assess relative risk (RR) for binary outcomes in cohort studies where outcomes were common, Poisson regression with robust variance was used [26]. The Cox proportional model’s hazard ratios (HRs) and 95% confidence intervals (CIs) were used to assess associations between predictive factors and time to medication discontinuation. The lower the hazard ratio, the lower the risk of medication discontinuation. All data collection and statistical analysis were performed using SAS 9.4 (SAS Institute, Cary, NC, USA). The level of significance was set at *p* < 0.05.

### 2.5. Sensitivity Analysis

Sensitivity analyses were performed to examine differences in adherence and persistence between the two groups. Adherence was measured as an MPR being 0.75 as a cut-off [27,28] and compared with the base case, which utilized an MPR of 0.80. Persistence was measured using a 14-day gap in treatment as a cut-off and compared with the base case, which utilized a 28-day allowable gap.

## 3. Results

During the pre- and post-policy periods, 85,521 and 91,494 patients were included, respectively. More than 60% of the study population was female and were started with SSRIs (selective serotonin reuptake inhibitor, Table 1). The gender and age distribution of the study population changed significantly, with the share of younger (under 35) and male patients increasing in the post-policy period.

Table 2 reports the number of IP sessions, medication adherence, and persistence by payment scheme. During the pre-policy period, the mean number of IP visits per person was 3.8 (SD = 3.85), while that number nearly doubled to 6.84 (SD = 7.72) during the post-policy period. Specifically, the proportion of the longer visits that lasted for more than 10 min increased.

Table 3 shows that the mean of LOT and MPR increased significantly from 36 days and 0.20 in the pre-policy period to 56 days and 0.33 in the post-policy period, respectively (*p* < 0.0001), and the trend was consistent in all age and gender subgroups. A similar trend was observed in the mean of the LOT.

Adherence defined by an MPR > 0.8 increased from 2538 (2.98%) pre-policy to 14,917 (16.3%) post-policy (RR = 5.48, 95% CI = 5.26–5.71) (Table 4). Even after adjusting for various confounding factors, patients in the post-policy group were still more likely to adhere to their medicine as prescribed than patients in the pre-policy group (adjusted RR = 4.06, 95% CI = 3.89–4.24, Table 4). The overall cohort had a mean (SD) time to non-persistence of 36.1 (41.31) days pre-policy and 55.6 (62.60) days post-policy, respectively (*p* < 0.0001, Table 2). A Cox proportional-hazards model showed that the probability of non-persistence was considerably reduced by 32% in the post-policy cohort, compared with the pre-policy (HR = 0.68, 95% CI = 0.67–0.68). After adjusting for baseline, persistence was affected more than other variables, but patients in the post-policy period were still more likely to persist and continue taking the medication than patients in the pre-policy period (adjusted HR = 0.85, 95% CI = 0.84–0.85).

**Figure 3:**
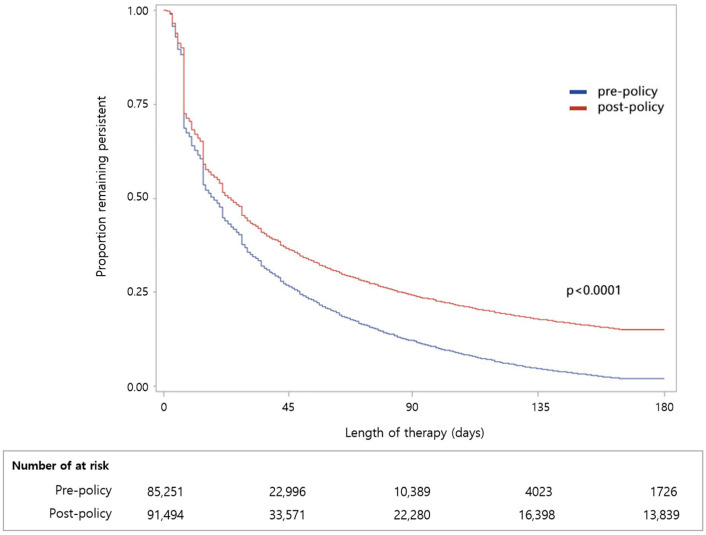
Time to discontinue antidepressant therapy for patients with depression for 180 days after initiation of individual psychotherapy.

Figure 3 shows Kaplan–Meier curves of the time to medication discontinuation for 6 months after IP. Approximately 30% of the patients in both groups discontinued their medication within the first 7 days, and the two groups diverged significantly after 14 days. The median time to discontinuation was 18 days pre-policy, and 25 days post-policy. The persistence was significantly longer post-policy than pre-policy (*p* < 0.0001).

The results of the sensitivity analyses were consistent with the base case analysis. Adherence measured at 0.75 MPR was slightly lowered (adjusted RR = 3.45, 95% CI = 3.33–3.59, data not shown), and still showed a significant increase in the post-policy period. Persistence measured with a 28-day allowable gap was also lowered (adjusted HR = 0.66, 95% CI = 0.65–0.67, data not shown).

## 4. Discussion

Combining psychotherapy and pharmacotherapy for patients with depression has been quite successful [29], and IP has been reported to increase medication adherence even in schizophrenia [30]. With the introduction of a new payment scheme, the goal of this study was to assess whether the medication adherence or persistence of patients diagnosed with depression improved as the number and length of IP sessions increased.

We analyzed the impact of the payment scheme change on medication adherence and persistence among patients with depression using nationally representative data. Our results showed that there was a significant improvement in both adherence and persistence in the post-policy period. A 6-month follow-up study showed that the adherence rate increased from 20 to 33%, and persistence increased from 36 to 56 days.

These findings are consistent with previous studies, which showed that the introduction Medicare Part D is associated with an improved adherence to and use of antidepressants in older adults with depression [31,32]. People with Part D coverage, no matter what kind of insurance they had before 2006, were much more likely to have an antidepressant adherence of 80% or greater (OR = 1.86 [95% CI: 1.44–2.39] for no coverage, 1.74 [95% CI: 1.25–3.42] for a USD 150 cap; and 1.19 [95% CI: 1.06–1.34] for the USD 350 cap groups) [31]. In addition to depression, several studies have shown that lowering cost-sharing enhanced medication adherence in other diseases [33,34,35]. We noticed that policy changes made it possible for physicians to give enough time for patient consultation, which could indirectly help people adhere to their medications, and thus lowering financial barriers to continuing treatment.

Although the frequency and quality of consultation have increased significantly after the implementation of the policy, given that IP sessions typically consist of 15 to 24 sessions lasting 45 to 60 min in the United States [36], this progress in Korea may not seem sufficient. However, it is necessary to understand the outpatient consultation environment and the trends of the treatment of depression in Korea. It is known that even if people experience depression due to a negative social perception in Korea, they tolerate it on their own rather than receiving counseling or treatment [37], and the proportion of those who actively receive treatment is only half of that of other developed countries [38]. In addition, several studies have identified the short consultation time in Korea [39,40,41]. Therefore, comparing the quantity and time of IP sessions with prior studies is difficult. However, the improvement in IP sessions seen in this study may have been an important factor in enhancing medication adherence and persistence.

Bultman et al. (2000) and Santana et al. (2011) have shown that belief in treatment is an important factor in improving medication adherence in patients with depression [42,43]. Because our results are for patients who have been treated with IP, we have difficulty comparing them with previous studies in psychotherapy. Instead, we compared trends of demographic characteristics. The results that the mean of the MPR and the LOT increased in patients who continued to receive IP and that females with depression have a higher percentage of adherence and persistence than men is similar to previous studies [44,45]. However, in terms of age groups, an opposite result was observed compared with previous work [11,22,23]. The studies of Olfson et al. (2006) and Akincigil et al. (2007) were measured using patient questionnaires, and in other antidepressant studies, the result of age was interpreted because several factors, such as education and stigma, could be reflected [3,44]. The long-term impacts of the COVID-19 pandemic have recently been observed, and Klimkiewicz et al. (2021) reported that the epidemic is a severe factor that can increase depression in people [46]. Further studies on the long-term, clinical characteristics of the patient and the presence or absence of IP are needed.

This study also supports adherence outcomes following conventional antidepressant therapeutic classes. Several studies have found that patients starting treatment with SSRIs or SNRIs have significantly higher adherence than patients starting with TCA [47,48]. The chance of staying on medication changed depending on the first therapeutic class offered. This shows how important the first type of therapy for depression can be.

Our study has some limitations. First, our study population was defined based on the reimbursement criteria of the IP session so that we could compare the difference between before and after the introduction of the policy. Thus, our study population is rather heterogeneous, which is a limitation of our study. Second, we could not determine whether the patients actually ingested the medication because the MPR indicates only the possession of medication [17]. There is a possibility of overestimating actual adherence because the patients were assumed to have used all obtained medication. Although we proposed an adjusted RR to account for overestimation due to other variables, caution is still required when interpreting compliance. Third, we defined patients based on the primary diagnosis. Therefore, the result of estimating the MPR and persistence may be according to such an operational definition [17]. Finally, because no clinical information was available in the claims database, we cannot explain how our study’s results were related to factors such as disease severity, stigma, individual therapy environment, and outcome. However, our study has the following strengths. The NHIS claims DB for the national population is representative data and can be objectively confirmed to measure the MPR. This is an analytical study of the effect of the payment change on IP treatment and depression in Korea of medication adherence and persistence. Our research results are a study to evaluate the effectiveness of the changed payment scheme and can be helpful as primary data when further implementing health policies related to patients diagnosed with depression.

## 5. Conclusions

Based on a retrospective examination of a large data set from insurance claims, we found improvements in medication adherence and persistence among patients with depression who received IP after the payment scheme change. After 6 months of IP for depression, overall medication adherence and persistence increased from 20 to 33% and from 36.1 to 55.6 days, respectively. In addition, the patients experienced more IP sessions, and in particular, the number of sessions of more than 10 min increased significantly. Our results suggest that a longer IP counseling trend and improved medication adherence and persistence is observed with the payment scheme change in Korea. A strategy of ongoing interest and support should be implemented so that patients with depression may actively participate in IP.

## Figures and Tables

**Table 1:** Demographic characteristics of patients diagnosed with depression by payment scheme.

Variable		Pre-Policy (*n* = 85,251)	Post-Policy (*n* = 91,494)	*p*-Value ^a^
Gender *n* (%)	Male	29,205 (34.3)	32,082 (35.1)	0.0004
	Female	56,046 (65.7)	59,412 (64.9)	
Age ^1^ *n* (%)	19–34	27,405 (32.2)	31,765 (34.7)	<0.0001
	35–49	22,506 (26.4)	23,822 (26.1)	
	50–64	19,433 (22.8)	20,237 (22.1)	
	Over 65	15,907 (18.6)	15,670 (17.1)	
Type of index medication ^2^ *n* (%)	SSRI	51,867 (60.8)	56,720 (62.0)	<0.0001
	TCA	5454 (6.4)	4494 (4.9)	
	SNRI	3091 (3.6)	3760 (4.1)	
	Poly	14,739 (17.3)	15,820 (17.3)	
	Other ^3^	10,100 (11.8)	10,700 (11.7)	

SSRI, selective serotonin reuptake inhibitor. TCA, tricyclic antidepressant. SNRI, serotonin–norepinephrine re-uptake inhibitors. ^a^ *p*-value obtained by Chi-square test. ^1^ Age classification based on WHO (World Health Organization). ^2^ First observation medication based on index date (poly = observation 2 or more medication classes based on index date). ^3^ Other includes Hyperici herba, Mirtazapine, Trazodone, Ademetionine, Bupropion, Agomelatine, Moclobemide, and Tianeptine.

**Table 2:** Number of IP sessions, medication adherence, and persistence by payment scheme.

Variable		Pre-Policy (*n* = 85,251)	Post-Policy (*n* = 91,494)	*p*-Value
Number of psychotherapy sessions ^1^ mean (SD)	Short	2.34 (3.11)	2.18 (3.42)	<0.0001 ^a^
	Intermediate	1.31 (2.01)	3.89 (5.50)	<0.0001 ^a^
	Long	0.15 (0.68)	0.77 (1.15)	<0.0001 ^a^
	Total	3.80 (3.85)	6.84 (7.72)	<0.0001 ^a^
Proportion of intermediate and long IP, mean (SD)		0.53 (0.45)	0.63 (0.41)	<0.0001 ^a^
Continuation psychotherapy, *n* (%)	Early drop ^2^	66,009 (77.4)	55,641 (60.8)	<0.0001 ^b^
	Continue	19,242 (22.6)	35,853 (39.2)	
MPR ^3^, mean (SD)		0.20 (0.22)	0.33 (0.33)	<0.0001 ^a^
LOT ^4^, mean (SD)		36.1 (41.31)	55.6 (62.60)	<0.0001 ^a^

^a^ *p*-value obtained by *t*-test. ^b^ *p*-value obtained by Chi-square test. ^1^ Pre-policy: Short < 15 min, 15 ≤ Intermediate < 45 min, Long ≥ 45 min. Post-policy: Short < 10 min, 10 ≤ Intermediate < 40 min, Long ≥ 40 min (Appendix A Table A1); ^2^ Early drop was defined as dropping out after 1–5 session. ^3^ MPR (medication possession ratio) = sum of days’ supply during the observation period/total observation period (0–1). ^4^ Length of therapy (LOT) was calculated as the number of days between the first prescription and the last prescription before therapy discontinuation (gap < 15 days) or 180 days.

**Table 3:** Mean MPR (medication possession ratio) and persistence of patients with depression.

Variable		MPR ^4^, Mean (SD)			LOT ^5^, Mean (SD)		
		Pre-Policy	Post-Policy	*p*-Value ^a^	Pre-Policy	Post-Policy	*p*-Value ^a^
*n*		85,251	91,494		85,251	91,494	
Gender	Male	0.19 (0.21)	0.32 (0.33)	<0.0001	35.44 (40.34)	54.93 (61.91)	<0.0001
	Female	0.20 (0.22)	0.33 (0.33)	<0.0001	36.51 (41.81)	56.03 (62.97)	<0.0001
Age ^1^	19–34	0.21 (0.22)	0.34 (0.33)	<0.0001	37.54 (42.43)	58.20 (63.22)	<0.0001
	35–49	0.20 (0.22)	0.34 (0.34)	<0.0001	36.30 (41.34)	58.30 (64.08)	<0.0001
	50–64	0.20 (0.22)	0.31 (0.33)	<0.0001	35.29 (40.75)	53.21 (61.87)	<0.0001
	Over 65	0.20 (0.21)	0.30 (0.32)	<0.0001	34.56 (39.90)	49.57 (59.40)	<0.0001
Type of index medication ^2^	SSRI	0.20 (0.22)	0.33 (0.33)	<0.0001	36.75 (41.52)	57.26 (63.12)	<0.0001
	TCA	0.17 (0.19)	0.25 (0.29)	<0.0001	29.87 (37.14)	41.46 (55.07)	<0.0001
	SNRI	0.20 (0.22)	0.33 (0.34)	<0.0001	36.70 (41.75)	57.46 (64.76)	<0.0001
	Poly	0.21 (0.23)	0.34 (0.34)	<0.0001	38.21 (42.63)	59.22 (64.29)	<0.0001
	Other ^3^	0.19 (0.21)	0.28 (0.31)	<0.0001	33.23 (39.81)	47.09 (57.82)	<0.0001
Total		0.20 (0.22)	0.33 (0.33)	<0.0001	36.1 (41.31)	55.6 (62.60)	<0.0001

SSRI, selective serotonin reuptake inhibitor. TCA, tricyclic antidepressant. SNRI, serotonin–norepinephrine re-uptake inhibitors. ^a^ *p*-value obtained by *t*-test. ^1^ Age classification based on WHO (World Health Organization). ^2^ First observation medication based on index date (poly = observation 2 or more medication classes based on index date). ^3^ Other includes Hyperici herba, Mirtazapine, Trazodone, Ademetionine, Bupropion, Agomelatine, Moclobemide, and Tianeptine. ^4^ MPR (medication possession ratio) = sum of days’ supply during the observation period/total observation period (0–1). ^5^ Length of therapy (LOT) is the time from the date of the first prescription of the index medication to the discontinuation of therapy (1–180 days).

**Table 4:** Poisson generalized linear model (GLM) and Cox proportional hazards regression: estimate of medication adherence and persistence.

Variable	Adherence ^4^		Persistence ^5^	
	RR (95% CI)	aRR (95% CI)	HR (95% CI)	aHR (95% CI)
**Policy**				
Pre-policy	REF	REF	REF	REF
Post-policy	5.48 ** (5.26–5.71)	4.06 ** (3.89–4.24)	0.68 ** (0.67–0.68)	0.85 ** (0.84–0.85)
**Gender**				
Female	REF	REF	REF	REF
Male	0.93 ** (0.91–0.96)	0.96 * (0.93–0.99)	1.01 * (1.00–1.02)	0.95 ** (0.93–0.99)
**Age ^1^**				
19~34	REF	REF	REF	REF
35–49	0.90 ** (0.87–0.93)	1.09 ** (1.04–1.13)	1.02 ** (1.01–1.03)	0.92 ** (0.91–0.93)
50–64	0.81 ** (0.78–0.85)	1.15 ** (1.10–1.21)	1.08 ** (1.06–1.09)	0.84 ** (0.83–0.85)
≥65	1.00 (0.98–1.05)	0.86 ** (0.83–0.89)	1.12 ** (1.11–1.14)	0.77 ** (0.75–0.77)
**Type of index medication ^2^**				
SSRI	REF	REF	REF	REF
TCA	0.50 ** (0.46–0.57)	0.65 ** (0.58–0.72)	1.29 ** (1.27–1.32)	1.10 ** (1.08–1.13)
SNRI	0.69 ** (0.64–0.76)	0.83 ** (0.76–0.90)	0.99 (0.96–1.02)	1.03 * (1.00–1.06)
Other ^3^	0.91 ** (0.85–0.98)	0.95 ** (0.89–1.03)	1.15 ** (1.13–1.16)	1.04 ** (1.03–1.06)
Poly	0.96 (0.89–1.04)	1.01 (0.93–1.09)	0.97 ** (0.96–0.99)	0.97 ** (0.96–0.98)

* *p* < 0.05, ** *p* < 0.01. RR, relative risk. aRR, adjusted relative risk. HR, hazard ratio. aHR, adjusted hazard ratio. CI, confidence interval. REF, reference. SSRI, selective serotonin reuptake inhibitor. TCA, tricyclic antidepressant. SNRI, serotonin–norepinephrine re-uptake inhibitors. ^1^ Age classification based on WHO (World Health Organization). ^2^ First observation medication based on index date (poly = observation 2 or more medication classes based on index date). ^3^ Other includes Hyperici herba, Mirtazapine, Trazodone, Ademetionine, Bupropion, Agomelatine, Moclobemide, and Tianeptine. ^4^ Adherent was defined as an event and values are given as relative risk (95% confidence intervals) and patients were considered adherent when MPR ≥ 0.80. ^5^ Persistence values are given as hazard ratios (95% confidence intervals) and patients were considered persistent when over 90 days (duration therapy).

## Data Availability

Restrictions apply to the availability of these data. Data was obtained from NHIS and are available at http://nhiss.nhis.or.kr with the permission of NHIS.

## Appendix A

**Table A1:** Revision of the resource-based relative value scale for individual psychotherapy fees.

	Pre-Policy		Post-Policy	
	Time/Session	RBRVS	Time/Session	RBRVS
Short	<15 min (NN011)	154.87	<10 min (NN001)	145.52
Intermediate	15 ≤ IP < 45 min (NN013)	292.19	10 ≤ IP < 20 min (NN002)	290.82
			20 ≤ IP < 30 min (NN003)	475.38
			30 ≤ IP < 40 min (NN004)	675.53
Long	≥45 min (NN012)	454.83	≥40 min (NN005)	895.83

RBRVS, resource-based relative value scale. IP, individual psychotherapy.

**Table A2:** Demographic characteristics of depression by payment scheme.

Class	Generic Name	WHO-ATC Classification Code
TCA (Tricyclic antidepressant)	Amitriptyline	N06AA09
	Amoxapine	N06AA17
	Clomipramine	N06AA04
	Imipramine	N06AA02
	Nortriptyline	N06AA10
SSRI (Selective serotonin reuptake inhibitor)	Fluoxetine	N06AB03
	Fluvoxamine	N06AB08
	Paroxetine	N06AB05
	Sertraline	N06AB06
	Citalopram	N06AB04
	Escitalopram	N06AB10
	Vortioxetine	N06AX26
SNRI (Serotonin and norepinephrine reuptake inhibitor)	Venlafaxine	N06AX16
	Milnacipran	N06AX17
	Duloxetine	N06AX21
	Desvenlafaxine	N06AX23
Other ^1^	Hyperici herba	N06AX25
	Mirtazapine	N06AX11
	Trazodone	N06AX05
	Ademetionine	A16AA02
	Bupropion	N06AX12
	Agomelatine	N06AX22
	Moclobemide	N06AG02
	Tianeptine	N06AX14

TCA, tricyclic antidepressant. SSRI, selective serotonin reuptake inhibitor. SNRI, serotonin–norepinephrine re-uptake inhibitors. ^1^ Other includes Hyperici herba, Mirtazapine, Trazodone, Ademetionine, Bupropion, Agomelatine, Moclobemide, and Tianeptine.
